# Transmission of SARS-CoV-2 by children: a rapid review, 30 December 2019 to 10 August 2020

**DOI:** 10.2807/1560-7917.ES.2022.27.5.2001651

**Published:** 2022-02-03

**Authors:** Barbara Clyne, Karen Jordan, Susan Ahern, Kieran A Walsh, Paula Byrne, Paul G Carty, Linda Drummond, Kirsty K O’Brien, Susan M Smith, Patricia Harrington, Máirín Ryan, Michelle O’Neill

**Affiliations:** 1 Health Information and Quality Authority, Dublin, Ireland; 2 Department of General Practice, RCSI University of Medicine and Health Sciences, Dublin, Ireland; 3 Department of Pharmacology & Therapeutics, Trinity College Dublin, Trinity Health Sciences, St James’s Hospital, Dublin, Ireland

**Keywords:** coronavirus disease 2019, SARS-CoV-2, transmission, children, coronavirus, schools

## Abstract

**Background:**

The role of children in the transmission of SARS-CoV-2 during the early pandemic was unclear.

**Aim:**

We aimed to review studies on the transmission of SARS-CoV-2 by children during the early pandemic.

**Methods:**

We searched MEDLINE, Embase, the Cochrane Library, Europe PubMed Central and the preprint servers medRxiv and bioRxiv from 30 December 2019 to 10 August 2020. We assessed the quality of included studies using a series of questions adapted from related tools. We provide a narrative synthesis of the results.

**Results:**

We identified 28 studies from 17 countries. Ten of 19 studies on household and close contact transmission reported low rates of child-to-adult or child-to-child transmission. Six studies investigated transmission of SARS-CoV-2 in educational settings, with three studies reporting 183 cases from 14,003 close contacts who may have contracted COVID-19 from children index cases at their schools. Three mathematical modelling studies estimated that children were less likely to infect others than adults. All studies were of low to moderate quality.

**Conclusions:**

During the early pandemic, it appeared that children were not substantially contributing to household transmission of SARS-CoV-2. School-based studies indicated that transmission rates in this setting were low. Large-scale studies of transmission chains using data collected from contact tracing and serological studies detecting past evidence of infection would be needed to verify our findings.

## Introduction

The coronavirus disease (COVID-19) pandemic is a global public health emergency, with over five million cases and over 1.5 million deaths worldwide at time of writing (December 2020) [1,2]. During the early pandemic, in the absence of effective treatments or vaccinations, efforts to reduce transmission were the most effective means of tackling the pandemic. Understanding the burden and transmission of severe acute respiratory syndrome coronavirus 2 (SARS-CoV-2) in children is essential for implementing appropriate public health measures and taking decisions about the provision of childcare and education. Adults and children of any age can be infected with SARS-CoV-2, however, paediatric SARS-CoV-2 infection is often milder, most children with symptomatic COVID-19 have a low risk of death or hospitalisation, and a large proportion are likely to be asymptomatic [3-5]. Initially, reported cases of COVID-19 in paediatric populations accounted for a small percentage of all diagnosed cases. International data suggested that children and young people accounted for 4% to 7% of reported cases [6,7]. Over time, the proportion of paediatric COVID-19 cases has risen as overall transmission has increased [8]. Fewer cases may have been initially diagnosed in children, leading to potential under-reporting because of initial test capacity constraints. There was an international trend towards restricting or prioritising real-time reverse transcription polymerase chain reaction testing to designated groups such as healthcare workers and or those with a greater risk of complications because of underlying disease. Potential under-reporting could have also been because of a reduction in typical age-mixing such as with school contacts [9,10].

There is growing evidence that children are less susceptible to severe SARS-CoV-2 infection than adults [11]. This may relate to differences in the way children interact with their environment and their dynamic developmental physiology and immunity [11]. Lower susceptibility appeared to be confined to those younger than 10–14 years old, while the risk in teenagers appeared to be closer to young adults [11]. A mathematical modelling study using data from six countries estimated that susceptibility to infection in individuals under 20 years of age was approximately half that of adults aged over 20 years [12]. The authors argued that low case rates in children and young people could also be explained by age-specific severity i.e. children are more likely to be asymptomatic and experience milder symptoms [12]. This may also represent under-testing and under-reporting in this age group in the early pandemic, as well as issues of potential lower diagnostic accuracy in asymptomatic cases. Many of the early pandemic studies were of low quality and data was limited [11].

Age-related transmissibility of SARS-CoV-2 has become an important public health concern, however, the role that children played in transmission during the early pandemic was unclear.

Our aim was to synthesise the available evidence on how children were contributing to the spread of SARS-CoV-2 during the early pandemic.

## Methods

Our team at the Health Information and Quality Authority (HIQA) of Ireland has conducted a series of rapid reviews for a broad range of public health topics related to COVID-19. These followed a standardised protocol developed a priori [13], in line with Cochrane rapid review methodology guidance [14]. The need for rapid reviews arose directly from questions posed by policymakers and expert clinicians supporting the National Public Health Emergency Team. The findings of these reviews have informed the national response to the COVID-19 pandemic in Ireland [15] and have implications for international health policy as well as clinical and public health guidance. Sections of this manuscript have been published on HIQA’s website as a white paper [16].

### Search strategy

Searches were conducted in MEDLINE, Embase, Cochrane Library, Europe PubMed Central and two preprint servers medRxiv and bioRxiv. The search terms and detailed search strategy are available in the protocol [13]. Reference lists of included articles were also searched. No language restrictions were applied. The search period was from 30 December 2019 to 10 August 2020.

### Inclusion and exclusion criteria

All potentially eligible papers identified through the search, including preprints, were exported to Endnote X8.2 (Thomson Reuters, New York, United States (US)) and screened for relevance by the review team (BC, KJ, SA, KAW, PB, PGC, LD, KKOB). Any study (regardless of design) that addressed the research question was included as per the inclusion and exclusion criteria presented in Table 1.

**Table 1:** Population, outcomes and study types (POS) framework for inclusion of studies on transmission of SARS-CoV-2 by children, 30 December 2019–10 August 2020

Population, outcomes and study types		
Population	Children (under the age of 18 years) with a laboratory-confirmed positive test for SARS-CoV-2; Subgroups of interest: asymptomatic vs symptomatic (mild, moderate, severe)^a^	
Outcome	Primary outcomes: Confirmed transmission of SARS-CoV-2 rates Proportion of which are household transmissions Mean time to transmission/symptoms onset	
Types of studies	Include: Any study that reports on transmission of SARS-CoV-2 by children	Exclude: Studies where SARS-CoV-2 was not confirmed with a laboratory test

COVID-19: coronavirus disease; SARS-CoV-2: severe acute respiratory syndrome coronavirus 2.

^a^ The disease severity classification by the World Health Organisation at time of writing: https://apps.who.int/iris/handle/10665/330893.

### Data extraction and quality assessment

For each included study, the study design, participant demographics and clinically relevant data including the numbers of children, index cases, infected contacts and contacts followed up were extracted.

The majority of studies included were case series and modelling studies. No universally accepted quality appraisal tool existed at the time of conducting this review for such study designs. Therefore, a series of questions adapted from related tools were used, as described in the protocol and the Supplement [13]. In view of study heterogeneity a meta-analysis was not possible and findings were synthesised narratively.

### Ethical statement

Ethical approval was not required as this was a review of published literature.

## Results

### Characteristics of included studies

A total of 28 studies were included, comprising 19 studies on household and close contact transmission [17-35], six on transmission of SARS-CoV-2 in educational settings [36-41] (Table 2) and three modelling studies estimating age-specific transmissibility of SARS-CoV-2 [42-44] (Table 3). Six studies were from China [17,18,22,23,25,42], three each from the US [27,29,32] and Israel [33,39,43], two from South Korea [28,40], and one report each was from Ireland [37], Switzerland [24], Australia [38], France [19], Italy [20], Vietnam [21], Singapore [45], the Netherlands [34], Thailand [26], Morocco [30], Finland [36], India [31] and New Zealand [44]. A secondary data analysis study included data from China, Singapore, South Korea, Japan and Iran [35]. Seven studies were preprints and had not undergone formal peer review at the time of writing [25,35,36,40,42-44]. Sample sizes for included COVID-19 cases in children ranged from 1 to 42,618, and where reported, contact numbers ranged from 1 to 57,415 in household and close contact transmission studies [19,24,26-29,31,33] and from 119 to over 10,000 in educational settings [36-41]. The age of participants (where reported) ranged from 32 days [20] to younger than 18 years [25]. From the 15 studies that reported demographic information, there was a total of 126 male and 104 female children [17-24,26,28-30,32,35,38].

**Table 2:** Characteristics of studies on transmission of SARS-CoV-2 by children, 30 December 2019–10 August 2020 (n = 25)

Author	Country	Study design	Population setting	Demographics		Primary outcome results
				Age	Sex	
**Household and close contact transmission**						
Cai et al. [17]	China	Case series	10 patients admitted to a children’s hospital for screening based on presentation with acute fever and/or respiratory symptoms and an epidemiological link to an adult case/exposure to an epidemic area	3–131 months old (mean: 74 months)	Male (n = 4); female (n = 6)	Confirmed transmission from infected child to adult contacts (n = 2); 3-month-old infant whose parents developed symptomatic COVID-19 7 days after looking after the infant; source of infant infection not reported
Canarutto et al. [20]	Italy	Case report	One patient admitted to a children’s hospital	32 days old	Male (n = 1)	Confirmed transmission from infected child to adult contacts (n = 0); no transmission of SARS-CoV-2 to medical staff was documented; transmission status to family members not reported
Danis et al. [19]	France	Case series (cluster)	One paediatric case and 172 contacts (112 school-based) of whom 73 had RT-PCR tests	9 years old	Male (n = 1)	Confirmed transmission from infected child (n = 0)
Jung et al. [28]	South Korea	Case report	One patient admitted to hospital with a 1-day history of headache; SARS-CoV-2 PCR-negative on admission; 81 close contacts and 1,125 casual contacts (219 inpatients with guardians, 81 discharged patients with guardians, 48 visitors and 858 healthcare workers)	9 years old	Female (n = 1)	Possible transmission from child to adult contact (n = 1); one of 1,152 contacts tested positive: mother of a hospitalised infant (22 days after birth) who shared the 6-patient room directly across from the index patient (ca 3 m distance); this mother had spent 2 h in the 6-patient room on 28 March 2020 and 20 h from 30 to 31 March 2020 (date on which index patient received positive test result)
Laxminarayan et al. [31]	India	Observational	42,618 index cases (aged 0–17 years) and 57,415 contacts with laboratory test results from public health surveillance data in Tamil Nadu and Andhra Pradesh states	0–4 years old (n = 5,624); 5–17 years old (n = 36,993)	Not reported	Confirmed transmission: positive contacts 0–4 years old: 460/7,341 (6%); 5–17 years old: 3,650/50,073 (7%)
Le et al. [21]	Vietnam	Case report	One patient admitted to a children’s hospital	3 months old	Female (n = 1)	Confirmed transmission from infected child to adult contacts (n = 0); at hospitalisation (day 6 after symptom onset) the infant was isolated with her mother; the infant’s mother was advised to wear a surgical face mask, practiced hand hygiene, and continued to breastfeed the infant; repeated maternal nasopharyngeal swabs were negative for SARS-CoV-2
Lin et al. [22]	China	Case report	One patient admitted to a quarantine ward in a local country hospital; RNA-positive throat swabs	7 years old	Female (n = 1)	Possible transmission from infected child to adult contacts: (n = 1); on 21 January 2020 the girl’s father drove and then took a bus to Xiangyang, Hubei province where he stayed overnight but did not have close contact with anybody except family members. On 22 January 2020, the father drove himself from Xiangyang, Hubei to Chongqing city with the girl, her grandparents, mother, and 2‐year‐old brother, arriving in the early morning of 23 January 2020
Lucar et al. [29]	US	Case report	One index case who was airlifted to hospital following a car accident and placed under general anaesthesia for orthopaedic surgical intervention tested SARS-Cov-2-positive on hospital day 2; 11 HCW present in the operating room	17 years old	Male (n = 1)	Confirmed transmission from child to HCW contacts (n = 0); 1/11 (assisted with intubation) developed a dry cough a few hours post-procedure but tested SARS-CoV-2 negative; 10/11 no symptoms throughout the 14-day monitoring period and were not offered SARS-CoV-2 testing; HCW wore COVID-19 personal PPE consisting of N95 mask, face shield, gowns, and gloves
Mannheim et al. [32]	US	Case series	64 paediatric (aged ≤ 17 years) laboratory-confirmed SARS-CoV-2 cases reported to the Chicago Department of Public Health; 15 households with transmission data	Median age: 11 (IQR: 7–16)	Male (n = 36); female (n = 28)	Confirmed transmission (15 households); households with child index case: 4/15; child-to-child: 2/15; child-to-adult: 2/15; number of secondary cases not reported
Nassih et al. [30]	Morocco	Case report	One laboratory-confirmed SARS-CoV-2 case	2 years old	Female (n = 1)	Confirmed transmission from infected child (n = 0); child cared for by mother for duration of illness (1 month) using airborne and contact precautions and mother remained PCR- and serology-negative
Posfay-Barbe et al. [24]	Switzerland	Case series	39 patients aged < 16 years with SARS-CoV-2 infection (seven inpatients, 32 outpatients) and 111 household contacts	Median age 11.1 (IQR: 5.7–14.5)	Male (n = 17); female (n = 22)	Possible transmission: cluster with a child developing symptoms before any other household contacts: 3/39; number of secondary cases: four (three mothers and one father)
Qiu et al. [23]	China	Case report	One patient admitted to a children’s hospital; RNA-positive nasopharyngeal and rectal swabs	8 months old	Male (n = 1)	Confirmed transmission from infected child to adult contacts (n = 0)
Somekh et al. [33]	Israel	Case series	13 family clusters (36 adults, 58 children); index case was the first case of the infection in the family	6 months–17 years old	Not reported	Confirmed transmission: cluster with child index case: 1/13 (14.5-year-old male); transmission from infected child to adult contacts not reported; transmission from infected child to child contacts not reported; number of secondary cases not reported
van der Hoek et al. [34]^a^	The Netherlands	Prospective observational study (surveillance data)	732 transmission pairs from Dutch National Institute for Public Health and the Environment national surveillance data	4–12 years old (n = 9); 20 years old and older (n = 31)	Not reported	Confirmed transmission from infected child to child (n = 2); the source in one of these cases was aged 15–20-years old
Wongsawat et al. [26]	Thailand	Case series	Three paediatric COVID‐19 cases and three adult caregivers	4–8-year-olds	Male (n = 2); female (n = 1)	Confirmed transmission from infected child to adult contacts (n = 0); one case was isolated with his grandfather who was also infected with COVID‐19, and two cases were isolated with healthy caregivers; during isolation, children and caregivers were advised to wash hands frequently, not share personal items and were provided surgical masks
Wu et al. [18]	China	Case series	74 paediatric SARS-CoV-2 cases admitted in a women’s and children’s hospital	≤ 3 months old (n = 7); 3–6 months old (n = 4); 6 months–1 year old (n = 5); 1–3 years old (n = 12); 3–10 years old (n = 31); over 10 years old (n = 15)	Male (n = 44); female (n = 30)	Confirmed transmission from infected child to contacts (n = 0)
Xu et al. [25] (preprint at the time of writing)	China	Observational	419 index patients and their 595 household secondary infections; index patient: first case patient and the only person in the household who returned home from Wuhan/other cities in Hubei Province; secondary cases: patients who had no known exposure to virus sources outside of the family	Not reported	Not reported	No case 15 years of age or younger who was infected by index patient (first case patient) was reported; three index patients were aged under 18 years and infected three secondary cases, one aged 0–17 years, one aged 18–49 years and one aged > 65 years
Zhu et al. [35] (preprint at the time of writing)	China, Singapore, South Korea, Japan, and Iran	Secondary data analysis of published data	31 household transmission clusters, 94 cases including 20 paediatric SARS-CoV-2 cases; setting: review of published literature and datasets between December 2019 and March 2020	3 months–10 years old (n = 20);	Male (n = 13); Female (n = 7)	Confirmed transmission: cluster with paediatric index case: 3/31^a^; number of secondary cases: 5; cluster with paediatric index case, assuming that asymptomatic children are being mistakenly overlooked as the index case in familial clusters: 6/28
**Educational settings-based transmission**						
Dub et al. [36] (preprint at the time of writing)	Finland (Helsinki)	Retrospective cohort study with nested household transmission study	Two COVID-19 cases (one paediatric and one staff) in two schools and 184 contacts; paediatric case: one child and 121 close contacts: 103 school contacts (96 pupils from four classes and eight school staff) and 18 sports training contacts (16 children and two adults); adult case: one staff member and 63 exposed persons: 52 pupils (two classes) and 11 staff members; close contacts: less than 2 m, for at least 15 minutes; household transmission study: contacts of secondary cases identified from adult case: (n = 33); close household contacts (n = 20); regular household contacts (n = 9); extended household contacts (n = 4)	12 years old	Not reported	Transmission: transmission from child (89/121 contacts tested, nasopharyngeal and serum specimens) (n = 0); transmission from staff (42/52 contacts tested, serum specimens): staff to child (n = 7); staff to staff (n = 1); household transmission study (seven cases and 29 contacts, serum specimens); possible transmission from child to contact (n = 2); number of secondary cases: 5
Heavy et al. [37]	Ireland	Case series	Six COVID-19 cases (three paediatric and three adult) with a history of school attendance and 1,155 contacts (1,025 school contacts, 130 other settings); paediatric cases: one primary school, two secondary school (905 school contacts, 84 other)	10–15 years old (n = 3)	Not reported	Confirmed transmission from infected child (n = 0)
Macartney et al. [38]	Australia (New South Wales)	Prospective cohort study	27 COVID-19 cases (12 children, 15 staff) and 1,448 (1,185 student contacts, 263 staff contacts) close contacts from 15 schools (10 high schools, five primary schools) and 10 ECEC settings; 633/1,448 (43.7%) had nucleic acid testing, or antibody testing; index case: first identified laboratory-confirmed case who attended the facility while infectious; primary case: initial infectious case or cases in that setting, and might or might not have been the index case; secondary case: close contact with SARS-CoV-2 infection (detected through nucleic acid testing or serological testing, or both), which was considered likely to have occurred via transmission in that educational setting; close contact: a person who had been in face-to-face contact for at least 15 min or in the same room for two hours with a case while infectious; high schools 12 primary cases (eight students, four staff) with 696 close contacts (600 students, 96 staff); primary schools five primary cases (one student, four staff) with 218 close contacts (179 students, 39 staff); ECEC 10 primary cases (three children, seven staff) with 534 close contacts (406 children, 128 staff)	High schools (n = 8): median 15 (range: 14–16); primary schools (n = 1): 10 ECEC (n = 3): median 2 (range: 2–3)	High schools (n = 8): male (n = 5); female (n =3); primary schools (n = 1): female (n = 1); ECEC (n = 3): male (n = 1); female (n = 2)	Positive cases: 18 secondary cases: 10 children, eight staff in 4/25 settings; cases in schools (n = 5): two secondary schools with two children and one staff case (child source); one primary school with one child case and one staff case (adult source); one ECEC setting (n = 13 cases): seven children, six staff (adult source); confirmed transmission all settings: child case to child contacts: 2/649; all settings: child case to staff contacts: 1/103; all settings, staff contacts to child case: 8/536; all settings, staff contacts to staff case: 7/160
Stein-Zamir et al. [39]	Israel	Case series	Two student COVID-19 cases in one high school (not epidemiologically linked) and 1,312 contacts (1,161 student, 151 staff)	Not reported	Not reported	Possible transmission: positive cases (n = 178); students: 153/1,161 (13.2%); staff: 25/151 (16.6%); COVID-19 rates were higher in junior grades (7–9) than in high grades (10–12); additionally, 87 confirmed cases among close contacts (siblings, recreational contacts and parents of students; family members of school staff) of the school’s cases
Yoon et al. [40] (preprint at the time of writing)	South Korea	Secondary analysis of press release data	45 paediatric cases in 40 schools and kindergartens (12 high schools, eight middle schools, 15 elementary schools, five kindergartens) and at least 10,903 contacts	4–5 years old (n = 5); 6–12 years old (n = 19); 13–15 years old (n = 8); 16–18 years old (n = 13)	Not reported	Confirmed transmission from infected child (n = 1); number of secondary cases (n = 2); 11 year old child in elementary school transmitted the SARS-CoV-2 virus to two other children
Yung et al. [45]	Singapore	Case series	Three SARS-CoV-2 cases (two paediatric and one adult) who attended two preschools and one secondary school and 119 contacts (42 from paediatric cases, 93 from adult case); close contacts (e.g. students from the same class) were placed under quarantine; non-close contacts were not quarantined and continued with classes SARS-CoV-2-positive from contact tracing following their exposures to adult family household members who were part of a community cluster	12 years old and 5 years old	Not reported	Confirmed transmission from infected child (n = 0)

CI: confidence interval; COVID-19: coronavirus disease; ECEC: early childhood education and care; HCW: healthcare workers; HR: hazard ratio; IQR: interquartile range; PPE: personal protective equipment; SARS-CoV-2: severe acute respiratory syndrome coronavirus 2; US: United States.

^a^ One case included here is also included in the case series by Cai et al. [17].

^b^ Article was translated using Google Translate.

**Table 3:** Characteristics of studies on transmission modelling of SARS-CoV-2 by children, 30 December 2019–10 August 2020 (n = 3)

Author	Country	Study design	Data source and model parameters	Demographics		Results
				Age	Sex	
Dattner et al. [43] (preprint at the time of writing)	Israel	Modelling study	637 households comprising 3,353 people in Bnei Brak; household size ranged from two to over 10; household inclusion criteria: at least two members, with all household members tested and at least one with PCR-confirmed COVID-19; model parameters data sources: discrete stochastic dynamic model with based on surveillance data, parameter estimates obtained by a maximum likelihood method, where the likelihood function is computed based on the stochastic model via simulations	0–19 years old (n = 1,544); over 20 years old (n = 1,809); clinical characteristics: PCR-confirmed COVID-19 1,510/3,353 (45%) 0–19 years old 512/1,544 (33%); over 20 years old 998/1,809 (55%);	Not reported	Transmission modelling: children, when infected, are somewhat less prone to infect others compared with adults, although the result is not statistically significant; the infectivity of children is estimated to be HR: 85% (95% CI: 65–110) relative to that of adults
James et al. [44] (preprint at the time of writing)	New Zealand	Modelling study	Confirmed and probable COVID-19 cases (n = 1,499), domestic cases (n = 924) (62%); imported cases (n = 575) (38%) from a completed outbreak; clinical characteristics: not reported; model parameters data sources: comprehensive dataset from a completed outbreak; reconstructed multiple instances of the transmission tree using a Monte-Carlo technique for cases missing potential index case or where there were multiple potential index cases	Under 10 years old (n = 35); 0–65 years old (n = 1,261); over 65 years old (n =172)	Not reported	Transmission: children infected fewer people on average and had a lower secondary attack rate compared with adults and the elderly; expected number of secondary infections caused by age group at no alert level (pre 25 March 2020); under 10 years old: 0.87; 0–65 years old: 1.49; over 65 years old: 1.51; expected number of secondary infections caused by age group at alert level 4 (post 25 March 2020); under 10 years: < 1; 0–65 years: < 1; over 65 years: > 1
Zhao et al. [42] (preprint at the time of writing)	China	Mathematical modelling	29 COVID-19 cases; 10 with history of exposure to Huanan seafood market; 19 without exposure; model parameters data sources: age group proportions, birth rate and death rate (Wuhan statistical yearbook); other parameters: literature	Not reported	Not reported	Model with four-age groups: highest transmissibility occurred between the age groups 15–44 years and 45–64 years, among those 65 years and older, or from 45–64 years to 65 years and older; lowest transmissibility occurred from age group 0–14 years to 15–44 years, or from 45–64 years to ≤ 14 years; model with five-age groups: highest transmissibility occurred between age group 25–59 years and ≥ 60 years, or among 25–59 years; lowest transmissibility occurred from age group 15–24 years to 25–59 years, or from age group 0–5 years to 6–14 years, or, to 15–24 years

COVID-19: coronavirus disease; SARS-CoV-2: severe acute respiratory syndrome coronavirus 2.

### Study quality

The 19 primary studies reporting on household and close contact transmission were generally of poor quality in terms of design, as there was a lack of detail on how cases were selected, what the criteria for testing contacts was, what testing was undertaken and how consistently testing was conducted across all contacts (see Figure and Supplement) [17-25,33,37,38].

**Figure:**
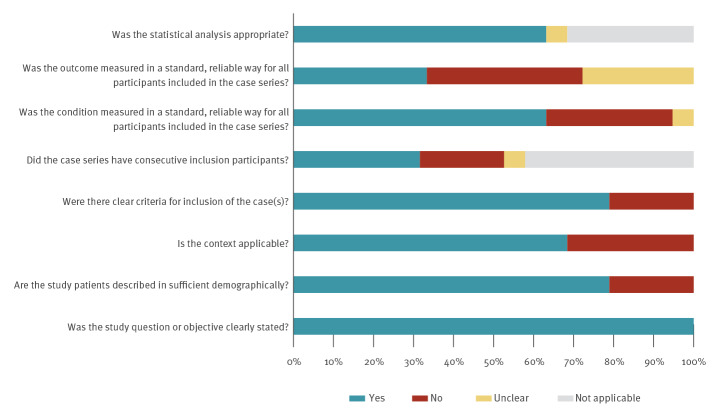
Methodological quality of included studies on transmission of SARS-CoV-2 by children, 30 December 2019–10 August 2020 (n = 28)

SARS-CoV-2: severe acute respiratory syndrome coronavirus 2.

Three studies had small sample sizes (less than 10) [17,19,26] and seven studies were case reports [20-23,28-30]. Two studies used existing public data [25,35], i.e. there is potential for double counting of cases across studies. In household studies, transmission was often assessed by self-reporting as to which family member developed symptoms first [24,32,33]. The number of contacts tested, the length of follow-up, which tests were performed and how long after the index case became positive, test sensitivity and specificity and confounders such as use of face masks and physical distancing were not consistently reported across studies. While household studies provide a unique opportunity to study transmission in a clearly identified cohort of close contacts, it is still possible that transmission events might be missed and errors in ascertaining the direction of transmission may be made. While all six studies in preschool and school settings had small numbers of cases who were children, they followed up a large number of contacts [36-41]. In three of these studies, only symptomatic contacts were followed and tested [37,38,41]. Given potential transmission from asymptomatic or mildly symptomatic contacts [3,4], the potential for missing cases cannot be ruled out. In all of these studies, household or community transmission cannot be ruled out for positive cases detected [38-40].

All three modelling studies used household data collected when countries were in various forms of lockdown and there were few child index cases [42-44]. The modelling study by Zhao et al. [42] had a small sample to check the fit of a model and the sample from the seafood market may not be representative of transmission patterns more generally. Data for the model by Dattner et al. [43] did not include dates of infection or information about who the index patient was. The study only used aggregate numbers of infected individuals in two age groups, 0–19-year-olds and 20-year-olds and older) and the majority of the data used were from households with two members. In James et al. [44] data on missing cases and missing transmission routes were estimated using Monte Carlo methods.

### Household and close contact transmission

Nineteen studies that examined household and close contact transmission comprised three analyses of surveillance data [27,31,34], eight case series [17-19,24,26,32,33,35], seven case reports [20-23,28-30] and one analysis of local heath commissions' public disclosures in China [25].

#### Children under 2 years of age

Four case reports which specifically included children under 2 years of age reported no transmission of SARS-CoV-2 [20,21,23,30]. In three case reports, no transmission from SARS-CoV-2-positive children aged 32 days to 2 years who were being cared for by their mothers was reported [21,23,30]. Use of precautions such as face masks was described in two of these studies [21,30]. In one case report, no transmission from a SARS-CoV-2-positive infant (aged 32 days) to medical staff was documented [20]. Details on personal protective equipment (PPE) and other precautions used by medical staff were not reported [20].

#### Children aged 0–10 years

Six studies included children aged 0–10 years [17,19,22,26,28,35], four of which reported transmission of SARS-CoV-2 from this age group. In a case series of 10 patients admitted to children’s hospitals in China, Cai et al. [17] reported transmission of SARS-CoV-2 from an infant aged 3 months to both parents, who developed symptomatic COVID-19 7 days after taking care of the infant. The authors of the study performing secondary analysis reported 31 SARS-CoV-2 household transmission clusters, of which three were identified as having a paediatric index case including the infant case described by Cai et al. [17] and two children aged 9 and 10 years [35]. In an analysis of these data, assuming a worst case scenario where asymptomatic children were the index case, six of the 28 family clusters would have a paediatric index case [35].

Two case reports also described transmission of SARS-CoV-2 by children in this age group [22,28]. A COVID-19 outbreak in a paediatric ward in South Korea was concluded to be a case of transmission from a 9-year-old child to an adult [28]. From 1,152 close and casual contacts tested, including the index patient's parents and healthcare workers, one contact had a positive SARS-CoV-2 PCR result. In a case report on a 7-year-old girl positive for SARS-CoV-2, Lin et al. [22], concluded that the girl infected her father. In both these cases, it is plausible that the source of infection was household or community transmission.

Two case series reported no transmission from children in this age group. In a case series of three paediatric COVID‐19 cases (aged 4–8 years) placed in isolation in a hospital in Thailand, one case was isolated with a grandfather co-infected with SARS-CoV-2, while two cases were isolated with their healthy caregivers [26]. Both healthy caregivers tested SARS‐CoV‐2- negative based on PCR tests of nasopharyngeal and throat swabs for the duration of the isolation, with no symptoms up to 14 days post discharge. Danis et al. [19] investigated a cluster of COVID-19 cases in the French Alps where a 9-year-old child with COVID-19, co-infected with other respiratory viruses (picornavirus and influenza A), visited three schools (duration of visits was not reported) and attended one ski class while symptomatic. From 172 contacts, no additional case of COVID-19 linked to the 9-year-old was identified.

#### Teenagers

Two studies specifically included teenagers. In one case report, no transmission from a SARS-CoV-2-positive 17-year-old to medical staff was documented following orthopaedic surgery [29]. An outbreak of SARS-CoV-2 at an overnight camp in Georgia, US, was detected after a teenage staff member (age not reported) tested positive for SARS-CoV-2 [27]. In total, test results for 344 of 597 camp attendees (aged 6–59 years) were available and 76% (260/344) were positive for SARS-CoV-2. Camp attendees engaged in a variety of indoor and outdoor activities (e.g. singing and cheering) and adherence to precautionary measures including physical distancing in cabins and use of face masks was not reported. As the authors highlight, given the increasing incidence of COVID-19 in Georgia at the time, household or community transmission cannot be ruled out for these cases, however, it is not clear whether these factors alone explain the high positivity rate observed [27].

#### Age unspecified

The remaining seven studies included children across a wide range of ages. Children younger than 16 years old were reported to be the index case in 8% of households (3/39, ages not specified) in Switzerland [24] and Israel (1/13 families, 14-year-old) [33]. Children aged 0–17 years were reported to be the index case in four of 15 households in Chicago, US [32], with two of the four household transmission clusters reported as child-to-child and two as child-to-adult [32]. In these studies, families were generally asked to self-report whether they developed symptoms before, after, or at the same time as the child case. Two of the three analyses of surveillance data documented transmission from children [31,34]. An analysis of 732 infector–infectee pairs conducted by the Dutch National Institute for Public Health and the Environment noted that COVID-19 was primarily spread between persons of approximately the same age [34]. Within the dataset, 23 of 31 cases aged under 20 years were infected in the home. Two of these 23 cases were categorised as child-to-child transmission although in one instance the source case was in the age group 15–20 years [34].

A study of public health surveillance data of confirmed cases and contacts in the high incidence setting of India, which included 42,618 children aged 0–17 years with 57,415 contacts, also found the highest probability of transmission within cases and contacts of a similar age [31]. A case series of 74 children with COVID-19 admitted to two hospitals in China reported no evidence that the virus was transmitted from these 74 children to others, although there is limited reporting on how this information was ascertained [18]. An analysis of local heath commissions' public disclosures data in China [25], based on 419 index patients and their 595 household secondary infections, reported no cases of infection by an index patient 15 years of age or younger. In a linked publication, the authors estimate that the hazard of being infected within households was higher for those aged under 18 years and those aged over 65 years, whereas the hazard of being infected outside of households was higher for those aged 18–64 years [46].

In summary, across these 19 household and close contact studies (where data was available), from a total of 42,926 reported children there were 42,639 cases where a child was considered to be the index case and 4,381 infected contacts (predominantly family members) were identified (Table 4). However, over 90% of these were from one study in a high incidence setting [31]. Where the specific age of index case was reported, transmission tended to occur from older children rather than from infants and young children. The total number of contacts could not be estimated and the majority of studies (10/19) did not report number of cases followed up. Where reported, contact numbers ranged from 1 to 57,415 [19,24,26-29,31,33] and the majority of contacts in those studies were not infected.

**Table 4:** Reported SARS-CoV-2 transmission by children in households and close contact transmission studies, 30 December 2019–10 August 2020 (n = 19)

Authors	Number of children included	Number of paediatric index cases^a^	Number of infected contacts	Number of contacts followed up
Children under 2 years of age				
Canarutto et al. [20]	1	0	0	Not reported
Le et al. [21]	1	0	0	Not reported
Nassih et al. [30]	1	0	0	1
Qui et al. [23]	1	0	0	Not reported
Children aged 0–10 years				
Cai et al. [17]	10	1	2	Not reported
Danis et al. [19]	1	0	0	172
Jung et al. [28]	1	1	1	1,206
Lin et al. [22]	1	1	1	Not reported
Wongsawat et al. [26]	3	0	0	2
Zhu et al. [35]	20	3	5	Not reported
Teenagers				
Lucar et al. [29]	1	1	0	11
Szablewski et al. [27]	1	1	260	597
Age unspecified				
Laxminarayan et al. [31]	42,618	42,618	4,110	57,415
Mannheim et al. [32]	64	4	Not reported	Not reported
Posfay-Barbe et al. [24]	39	3	4	111
Somekh et al. [33]	58	1	Not reported	94
Van Der Hoek et al. [34]	31	2	2^b^	Not reported
Wu et al. [18]	74	0	0	Not reported
Xu et al. [25]	Unclear^c^	3	3	Not reported
**Total**	**42,926**	**42,639**	**4,381**	**Not estimable**

SARS-CoV-2: severe acute respiratory syndrome coronavirus 2.

^a^ Some cases included may be double counted across studies.

^b^ The source in one of these cases was aged 15–20 years.

^c^ Unclear how many children included overall.

### Educational setting transmission

Six studies assessed transmission within educational settings [36-41]. All six studies included children in secondary school settings [36-41], while three also included children in preschool settings [38,40,41] and three additionally included children in primary school settings [37,38,40]. Three of six studies reported transmission of SARS-CoV-2 by children [38-40].

A study from New South Wales, Australia examined SARS-CoV-2 transmission in 25 educational settings, 15 primary and secondary schools, and 10 early childhood education and care settings (ECEC) over a 10-week period (25 January 2020–9 April 2020 with distance learning encouraged statewide in March) [38]. They identified 27 confirmed cases, 12 children and 15 staff members who attended school or ECEC settings while considered infectious, defined as 24 h before symptom onset, and 1,448 close contacts (1,185 students and 263 staff). In total, 633 of 1,448 close contacts had nucleic acid or antibody testing, with 18 secondary cases (10 children and eight adults) identified. Three secondary cases (one adult, two children) were linked to transmission from three children of secondary school age, resulting in an overall secondary attack rate of 0.3% and a secondary attack rate for child to staff member of 1.0%.

Based on analysis of data reported in press releases on 45 paediatric patients with COVID-19 in 40 schools and kindergartens, and 10,903 tested contacts by the Korean Centers for Disease Control and Prevention, Yoon et al. [40] reported one case where an 11-year-old child transmitted the virus to two other children. One child was infected in the same classroom and the other child was infected at the same gym outside the school setting. In Israel, a large outbreak was reported 10 days after schools reopened [39]. Following diagnosis of two unrelated cases, the entire school community i.e. 151 staff members and 1,161 students, was tested; 178 (25 staff 16.6%), 153 students (13.2%) tested positive for SARS-CoV-2. During this time, there was an extreme heat wave, with school attendees exempt from mask wearing, the air conditioning was continuously on and physical distancing could not be observed in crowded classrooms, which may have contributed to the spread of the virus.

The remaining three studies in educational settings reported no transmission of SARS-CoV-2 by children. An analysis of Irish notifications of SARS-CoV-2 in the school setting, before universal school closure on 12 March 2020 and introduction of public health measures, found no transmission from children [37]. Three paediatric cases (all aged 10–15 years) and three adult cases of COVID-19 were identified. The three paediatric cases had a total of 822 child and 83 adult contacts within the school setting. Contacts were exposed at school in the classroom and during school-related activities such as sports lessons, music lessons and choir practice. No additional cases were identified during the follow-up period of 14 days from last contact with the index case. However, only contacts who developed symptoms were referred for testing, thus asymptomatic secondary cases were not captured. Similarly, a report of SARS-CoV-2 transmission among children in two preschools and one secondary school in Singapore found no transmission from children [45]. At the time, schools in Singapore were not routinely closed, but targeted public health measures including deep cleaning of the schools and suspension of extracurricular activities were implemented. From a child index case aged 5 years in one preschool, a total of 34 preschool student contacts developed symptoms post-exposure and were tested for SARS-CoV-2, but SARS-CoV-2 was not detected. From a secondary school child index case aged 12 years, a total of eight students developed symptoms and were screened for SARS-CoV-2 during the incubation period, all of whom tested negative [45]. A report of SARS-CoV-2 transmission among children in two schools in Finland also found no transmission from children [36]. Exposure in school because of a 12-year-old index case who attended with mild symptoms led to no further cases among 89 of 121 close contacts tested.

In summary, across these six studies, there were 65 cases where a student was considered to be the index case and 183 additional infected contacts were identified from 14,003 close contacts (Table 5). There was no transmission from a child reported in the pre-school setting and one case where a primary school child was considered to be the index case (with two additional infected contacts). In the majority of cases, transmission from a child was reported in the secondary school setting. There were 35 secondary school index cases with 183 additional infected contacts, although the majority of these (97%) were from one study [39]. However, household or community transmission cannot be ruled out for these cases.

**Table 5:** Reported SARS-CoV-2 transmission by children in school-based studies, 30 December 2019–10 August 2020 (n = 6)

Author	Number of paediatric index cases	Number of contacts followed up	Number of infected contacts from paediatric index cases
**Pre-school (aged 0–5 years)**			
Macartney et al. [38]	3	122	0
Yoon et al. [40]	5	670	0
Yung et al. [41]	1	34	0
**Primary school (aged 5–11 years)**			
Macartney et al. [38]	1	45	0
Heavy et al. [37]	1	905^a^	0
Yoon et al. [40]	19	3,524^b^	2
**Secondary school (aged 12 years or older)**			
Dub et al. [36]	1	89	0
Heavy et al. [37]	2	905^a^	0
Macartney et al. [38]	8	586	3
Stein-Zamir et al. [39]	2	1,312	178
Yoon et al. [40]	21	6,709^b^	0
Yung et al. [41]	1	8	0
**Total**	**65**	**14,003**	**183**

SARS-CoV-2: severe acute respiratory syndrome coronavirus 2.

^a^ Number of contacts followed up by age of index case is not presented; number of contacts only counted once in overall total in this table.

^b^ 1,071 individuals were tested because of exposures from two brothers in the same family, one from a primary school and one from a secondary school setting. These 1,071 individuals have only been added to the primary school total.

### Transmission modelling

All three modelling studies estimated lower transmission in children compared with adults [42-44]. In a mathematical modelling study (using an adapted version of the of Susceptible-Exposed-Infectious-Recovered (SEIR) model) estimating age-specific transmissibility of SARS-CoV-2, Zhao et al. [42] concluded that SARS-CoV-2 had high transmissibility among adults aged 25 years or older, but low transmissibility among children younger than 14 years of age. The model fit was based on data from 29 cases, 10 of whom had exposure to the Huanan seafood market. Dattner et al. [43] fitted a dynamic stochastic age-of-infection model to estimate household SARS-CoV-2 transmission in Bnei Brak, Israel. Data were collected from 637 households (n = 3,353) of at least two members, with all household members tested and at least one with PCR-confirmed COVID-19. Transmission was estimated to be lower in children and young people (aged 0–19 years), compared with adults (aged 20 years or older), although this difference was not statistically significant (hazard ratio: 85%; 95% confidence interval: 65–110). James et al. [44], analysed the transmission dynamics of COVID-19 using a comprehensive dataset for a completed outbreak (at the time) in New Zealand. They estimated that although children younger than 10 years old were equally likely to infect at least one person, they contributed less to the spread of SARS-CoV-2 than other age groups, infecting fewer people on average and having a lower secondary attack rate.

## Discussion

The evidence in this review highlights that child-to-child and child-to-adult transmission of SARS-CoV-2 can occur. However, unlike in other viral epidemics, for example influenza [47], during the early stages of the COVID-19 pandemic, children did not appear to be key transmitters of SARS-CoV-2 with reported transmission from children being low. Studies published subsequent to this review have highlighted that the proportion of paediatric COVID-19 cases has risen as overall transmission has increased [5]. The majority of included studies have looked at household transmission related to known cases and it was often unclear who the index patient was. These results are consistent with similar previous reviews in the area [48,49]. In these studies, children aged 10 years and older may spread the virus more easily to family members than younger children and might even spread it as easily as adults [50]. Although not all studies presented a breakdown of children’s age, our results are consistent with this finding, with more cases of transmission linked to older children than younger children. However, these results should be interpreted in the context of the limitations of included studies.

Ten of the 19 included studies on household and close contact transmission reported child-to-child or child-to-adult transmission. Overall, from 19 studies, with a total of 42,926 reported children, 42,639 cases where a child was considered to be the index case and 4,381 infected contacts, predominantly family members, were identified. Four household transmission studies reported that children were the index case in 8% to 26% of households [24,32,33,35]. Zhu et al. [35] reported that even if the assumption was made that asymptomatic children were the index case in all familial clusters, it would still only produce a situation where children accounted for roughly 20% of household cluster transmissions. All three studies examining large scale surveillance data reported transmission from children at varying levels [27,31,34]. Two of these studies highlight that SARS-CoV-2 is primarily spread between persons of approximately the same age and the probability for contacts to have a positive test result is not clearly associated with their age [34]. Across the remaining case series and case reports, transmission was variable with a lack of information and inconsistencies of reporting within the manuscripts. In at least three studies that suggested transmission from children to other family members occurred [17,22,28], it is also feasible that the virus was transmitted from the parents or adults to the children. To date, published studies indicate that children are mostly infected by family members in the home [51-55]. Many SARS-CoV-2 clusters have been linked to a wide range of mostly indoor settings such as households and few reports came from schools [56]. This is perhaps unsurprising as many of the included studies were conducted in the context of strict physical distancing policies including school closures. Analysis of social contact and age-mixing patterns in China during the outbreak period, where strict physical distancing policies were in place, highlights that typical features of age-mixing (school contacts, workplace contacts and so on) decreased and contacts during the outbreak mostly occurred at home with household members [9,10].

Three included case reports [20,28,29] reported no transmission from children to healthcare workers. One study described the use of PPE consisting of N95 mask, face shield, gowns, and gloves [29], but the other studies did not provide any details. The risk of occupational exposure to SARS-CoV-2 may be lower in paediatric healthcare settings, because of a lower number of COVID-19 patients admitted in this setting and to higher awareness and compliance with protective measures [57].

Following a survey of the experience of 15 European countries related to the role of childcare and school settings in COVID-19 transmission in July 2020, and a review of the literature, the European Centre for Disease Prevention and Control (ECDC) reported few large-scale outbreaks of COVID-19 in schools [7]. The results of our review, which includes one additional new study and one updated study with data beyond the ECDC report, are in line with these findings. Across studies on transmission of SARS-CoV-2 in schools included in our review, there were 66 cases where a student was considered to be the index case and 183 additional infected contacts were identified from 14,513 close contacts. The majority of these cases (178/183) were identified in one study from Israel [39], where precautionary measures, such as mask wearing and physical distancing, were not observed and where household or community transmission cannot be ruled out for the cases. Studies published after the last date of our search, have highlighted that transmission in childcare settings may occur, but at low levels [58-60]. Studies from Germany, Italy and England have also reported that outbreaks have been uncommon or small since the reopening of schools began [61-64].

A rapid review on school closure during coronavirus outbreaks, including COVID-19, found limited and conflicting information [65]. The authors cite modelling studies of COVID-19 which predict that school closures alone would prevent 2% to 4% of deaths, much less than other physical distancing measures [65]. Subsequent modelling studies also report that school closures alone have limited impact on reducing the burden of COVID-19 [66,67]. The ECDC report also concluded closures of childcare and educational institutions are unlikely to be an effective single control measure for community transmission of COVID-19 [7]. However, school closures may have negative impacts for children including interruption of learning, mental health issues and exacerbation of disparities [7]. A systematic review conducted up to July 2021 also concluded that transmission of SARS-CoV-2 was markedly lower in school compared with household settings, suggesting that household transmission is more important than school transmission, particularly in the early pandemic [68].

All three modelling studies estimated lower transmission in children; however, there were few child index cases included across studies [42-44].

Although studies of real-life transmission remain limited, transmission potential of SARS-CoV-2 by children is influenced by a number of factors other than susceptibility to infection, including potential for exposure to the virus and viral load (i.e., the amount of virus that a child might carry). In relation to exposure to the virus outside of the home, a number of studies looking for evidence of past infection in childcare and school settings have been conducted. While these studies do not present evidence on actual transmission chains from infected children to others, they do characterise the burden of COVID-19 within these settings during the epidemic period. Across five studies, SARS-CoV-2 seroprevalance ranged from 0.7% to 39% for pupils and 0.2% to 43% for teachers [69-73]. A study in Belgian day care settings, undertaken shortly after the start of the local epidemic and before lockdown commenced, noted that while cold symptoms were common, RT-PCR tests of nasopharyngeal specimens taken from a random sample of children (n = 84) attending eight different day care centres were all negative for SARS-CoV-2 [74]. A study from January 2020 to March 2021 found that seropositivity in Israeli children aged 0–15 years was 1.8–5.5 times higher than COVID-19 incidence rates based on PCR testing [75].

Reliable, large-scale data on transmission from symptomatic and asymptomatic children are lacking. Large-scale studies focusing on transmission chains using data collected from contact tracing and case investigations would be needed to determine how children contributed to the spread of SARS-CoV-2. As schools and childcare facilities gradually reopened after summer 2020 worldwide, more data on chains of transmission linked to children outside of the household setting should be available. Serological studies looking for past evidence of infection, and studies assessing viral load in infected children and the relationship between viral load and transmission may also be helpful in understanding the role children play in transmission [76]. While we have adhered to fundamental methodological principles, in keeping with international rapid review methodology guidance [14,77], our study has some limitations. For study designs where no universally accepted quality appraisal tool existed (e.g. case series, modelling studies), a series of questions adapted from related tools were used, as described in the protocol and Supplement but it was not a validated tool [13]. Studies of household transmission which describe presumed contact and transmission, but did not specifically report transmission from child to adult [78] or the age of the index cases [79] were not included in this review. The initial search strategy was used to retrieve research relevant to COVID-19 specifically to address a number of review questions at the same time, therefore a detailed flow diagram was not kept as titles and abstracts were screened against a number of research questions.

## Conclusion

During the early pandemic there was limited and poor quality information on the contribution of children to the transmission of SARS-CoV-2. Few definitive cases of virus transmission from children had been published by the beginning of August 2020, with no clear evidence suggesting a higher rate of transmission from children than adults. The published studies identified, indicated that children were not disproportionately contributing to the household transmission of SARS-CoV-2 during the early pandemic, however, further high quality studies would be needed.

## Supplementary Data

599000 Supplement
