# Supplementary material for: Transmission of SARS-CoV-2 by children: a rapid review, 30 December 2019 to 10 August 2020
Source: Euro Surveill. 2022 Feb 3;27(5):2001651. doi: 10.2807/1560-7917.ES.2022.27.5.2001651 (PMC8815097; doi:10.2807/1560-7917.ES.2022.27.5.2001651)
Supplement: Supplement [file 20-01651_CLYNE_Supplement.pdf]

Supplementary material

This supplementary material is hosted by *Eurosurveillance* as supporting information alongside the article “Transmission of SARS-CoV-2 by children: a rapid review, 30 December 2019 to 10 August 2020”, on behalf of the authors, who remain responsible for the accuracy and appropriateness of the content. The same standards for ethics, copyright, attributions and permissions as for the article apply. Supplements are not edited by *Eurosurveillance* and the journal is not responsible for the maintenance of any links or email addresses provided therein.

Table 1 Methodological quality of included studies on transmission of SARS-CoV-2 by children, 30 December 2019–10 August 2020

	Household studies																			School						Model studies		
**Question**	**Cai**	**Canarutto**	**Danis**	**Jung**	**Laxminarayan**	**Le**	**Lin**	**Lucar**	**Mannheim**	**Nassih**	**Posfay-Barbe**	**Qiu**	**Somekh**	**Szablewski**	**van der Hoek**	**Wongsawat**	**Wu**	**Xu**	**Zhu**	**MACCARTHY**	**Heavey**	**Yung**	**YOON**	**Stein-Zamir**	**Dub**	**Dattner**	**James**	**Zhao**
Was the study question or objective clearly stated?	Yes	Yes	Yes	Yes	Yes	Yes	Yes	Yes	Yes	Yes	Yes	Yes	Yes	Yes	Yes	Yes	Yes	Yes	Yes	Yes	Yes	Yes	Yes	Yes	Yes	Yes	Yes	Yes
Are the study patients described in sufficient demographically?	Yes	Yes	No	Yes	Yes	Yes	Yes	Yes	Yes	Yes	Yes	Yes	No	Yes	Yes	Yes	Yes	No	Yes	No	No	No	No	No	Yes	No	No	n/a
Is the context applicable?	No	Yes	Yes	Yes	Yes	No	No	Yes	Yes	No	Yes	No	Yes	Yes	Yes	Yes	No	Yes	Yes	Yes	Yes	Yes	Yes	Yes	Yes	n/a	n/a	n/a
Were there clear criteria for inclusion of the case(s)?	Yes	No	Yes	Yes	Yes	Yes	Yes	Yes	Yes	Yes	Yes	No	No	Yes	Yes	Yes	Yes	Yes	No	Yes	Yes	Yes	Yes	Yes	Yes	Yes	Yes	Yes
Did the case series have consecutive inclusion participants?	No	n/a	Yes	n/a	n/a	n/a	n/a	n/a	Yes	n/a	Yes	n/a	n/a	Yes	Unclear	Unclear	Yes	No	No	No	No	No	Yes	Yes	Yes	n/a	n/a	n/a
Was the condition measured in a standard, reliable way for all participants included in the case series?	Yes	Unclear	No	Yes	No	Yes	Yes	No	Yes	Yes	Yes	Yes	Yes	No	Yes	Yes	No	Yes	No	No	Yes	Yes	Yes	Yes	Yes	Yes	No	n/a
Was the outcome measured in a standard, reliable way for all participants included in the case series?	Yes	Unclear	No	Yes	No	Unclear	Unclear	No	Unclear	Yes	No	Unclear	Yes	Yes	Yes	Yes	No	Yes	No	No	No	Yes	Yes	Yes	Yes	n/a	n/a	n/a
Was the statistical analysis appropriate?	Yes	n/a	Yes	n/a	Yes	n/a	No	n/a	Yes	n/a	Yes	n/a	Unclear	Yes	Yes	Yes	Yes	Yes	Yes	Yes	Yes	Yes	Yes	Yes	Yes	Modelling study - findings based on paraemter estimates using a discrete stochastic dynamic model of the spread of the epidemic within a household and data from the Israeli COVID-19 database	Modelling study - findings based on a Monte-Carlo network construction techniques (using comprehensive outbreak dataset) to provide an estimate of the number of secondary cases for every individual infected during the outbreak.	Modelling study - findings were based on parameter estimation and data from literature
